# Impact of cardiometabolic health on treatment outcomes in early-stage triple-negative breast cancer receiving chemoimmunotherapy

**DOI:** 10.1007/s10549-026-07995-5

**Published:** 2026-06-12

**Authors:** Jasmin Hundal, Asfand Cheema, Akram Abushamma, Kirti Arora, Xiaoying Chen, Kelly Fargo, Maiti Baidehi

**Affiliations:** 1 Department of Hematology and Medical Oncology, Taussig Cancer Center, Cleveland Clinic, Cleveland, OH USA; 2 Department of Internal Medicine, Cleveland Clinic, Cleveland, OH USA; 3 Department of Internal Medicine, Cleveland Clinic Akron General, Akron, OH USA; 4 Department of Quantitative Health Science, Cleveland Clinic, Cleveland, OH USA; 5 Department of Pharmacy, Cleveland Clinic, Cleveland, OH USA; 6 Medical Oncology and Hematology Team Lead, Breast Medical Oncology, West Region Faculty, Internal Medicine Residency Program, & MS4 Clinical Education, Fairview Hospital, Ohio University Cleveland Clinic Cancer Center at the Fairview Hospital, 18200 Lorain Ave, Cleveland, OH 44111 USA

**Keywords:** Triple-negative breast cancer, Pembrolizumab, KEYNOTE-522, Metabolic syndrome, Cardiometabolic comorbidities, Pathologic complete response, Overall survival, Hypertension

## Abstract

**Background:**

Triple-negative breast cancer (TNBC) lacks effective targeted therapies, and pivotal trials of pembrolizumab with neoadjuvant chemotherapy (KEYNOTE-522) underrepresent patients with cardiometabolic comorbidities. We evaluated how metabolic dysfunction affects treatment tolerability, pathologic complete response (pCR), and overall survival (OS) in routine care.

**Methods:**

We conducted a retrospective cohort study of women with early-stage triple-negative breast cancer (TNBC) treated with the KEYNOTE-522 regimen at the Cleveland Clinic between January 1, 2020, and March 31, 2025. Metabolic syndrome was defined using a constellation of cardiometabolic risk factors consistent with criteria proposed by the American Heart Association and the National Heart, Lung, and Blood Institute, adapted to available real-world clinical data (BMI, HbA1c in place of fasting glucose, hypertension, chronic kidney disease, HDL < 50 mg/dL, triglycerides > 150 mg/dL; ≥3 criteria). Data included demographics, tumor characteristics, treatment details, cardiometabolic comorbidities, CTCAE-graded adverse events, pathologic complete response (pCR), and overall survival (OS). Kaplan–Meier methods estimated survival; logistic regression evaluated predictors of pCR; Cox proportional hazards models assessed OS. Median follow-up was 28.8 months.

**Results:**

Among 222 patients with early-stage TNBC treated with the KEYNOTE-522 regimen, 55 (24.8%) met criteria for metabolic syndrome. Patients with metabolic syndrome were older (median 65 vs. 55 years, *p* = 0.0004) and more frequently Black (43.6% vs. 18.0%). Cardiometabolic comorbidities were more prevalent, including obesity (85.5% vs. 35.9%), diabetes (72.7% vs. 7.8%), hypertension (69.1% vs. 24.6%), CKD (21.8% vs. 4.8%), low HDL (83.6% vs. 12.0%), and elevated triglycerides (58.2% vs. 6.0%) (all *p* < 0.001). Dose reductions were more frequent among patients with metabolic syndrome (32.7% vs. 20.4%), with similar treatment discontinuation rates. pCR rates did not differ significantly (72.7% vs. 68.9%, *p* = 0.62), and no cardiometabolic factors independently predicted pCR. With median follow-up of 28.8 months, metabolic syndrome was associated with worse overall survival. Hypertension (HR 3.83, 95% CI 1.47–9.96, *p* = 0.006) and diabetes (HR 3.07, 95% CI 1.33–7.08, *p* = 0.009) were independent predictors of mortality.

**Conclusion:**

Cardiometabolic comorbidities, particularly hypertension, were associated with poorer survival and greater treatment intolerance despite preserved pCR. Systematic cardiometabolic assessment and proactive management should be integrated into TNBC care.

**Supplementary Information:**

The online version contains supplementary material available at 10.1007/s10549-026-07995-5.

## Introduction

Breast cancer (BC) remains a significant global health burden, with an estimated 313,510 new cases in the year 2024 in the US, and over 99% of cases in women [1]. Triple-negative breast cancer (TNBC) is defined by the absence of estrogen Receptor (ER), progesterone Receptor (PR), and Human Epidermal Growth Factor Receptor 2 (HER-2). The mean annual incidence of TNBC is 13.7 per 100,000 women, with higher rates among African American women [2]. TNBC is clinically heterogeneous and exhibits variable treatment responses and lacks targeted therapies, leading to poor outcomes [3–5].

Historically, chemotherapy is the primary treatment for TNBC [6]. The biological profile of TNBC, including high levels of tumor-infiltrating lymphocytes (TIL) and programmed cell death Ligand (PDL1) expression, has provided a rationale for incorporating immune checkpoint inhibitors (ICIs) into treatment regimens [6–10].

In the KEYNOTE-522 trial, adding pembrolizumab to neoadjuvant chemotherapy improved pathologic complete response (pCR) rates (64.8% vs. 51.2%) and event-free survival (84.5% vs. 76.8%) compared with chemotherapy alone [11, 12]. However, clinical trial populations typically exclude patients with significant comorbidities, limiting generalizability. Real-world data on how cardiometabolic disorders affect the efficacy and survival of chemoimmunotherapy remain scarce.

While ICIs improve outcomes, they are associated with immune-related adverse events (iRAEs) affecting multiple organ systems. Metabolic toxicities, such as thyroid dysfunction, diabetes, and other endocrine or nutritional disorders, are increasingly recognized but remain underreported [13–15]. Furthermore, chemotherapy and immunotherapy may exacerbate pre-existing cardiometabolic comorbidities, which can influence both treatment tolerability and long-term outcomes.

Despite growing trial evidence, real-world data on TNBC patients receiving pembrolizumab-based neoadjuvant therapy are limited, particularly regarding how comorbidities such as metabolic syndrome, hypertension, diabetes, and chronic kidney disease impact treatment response and survival. Real-world studies provide external validity to trial findings and can identify subgroups at higher risk for adverse outcomes [16].

This study evaluated early-stage TNBC patients treated with neoadjuvant pembrolizumab plus chemotherapy according to the KEYNOTE-522 regimen at a large academic center. We examined cardiometabolic comorbidities, treatment tolerability, and clinical outcomes, including pCR and OS, to better understand real-world prognostic factors in this population.

## Methods

We conducted a retrospective cohort study of early-stage triple-negative breast cancer (TNBC) patients treated with the Keynote-522 regimen at the Cleveland Clinic, Cleveland, Ohio, between January 1, 2020, and March 31, 2025. The Institutional Review Board approved the study. Eligible patients were identified from institutional records.

Female patients aged ≥ 18 years with histologically confirmed early-stage TNBC who received neoadjuvant chemotherapy as per the Keynote-522 regimen were included. Patients with breast cancer subtypes other than TNBC and those under 18 years of age were excluded from the study.

Data were retrospectively extracted from the electronic medical records. They included patient demographics (age, race, body mass index [BMI]), clinical variables (tumor stage), genetic testing results (BRCA1/2 mutation status), and smoking status. Pharmaceutical management for metabolic co-morbidities was also collected (Supplementary Table 3).

Cardiometabolic profiles were comprehensively characterized using a metabolic syndrome constellation approach consistent with criteria proposed by the American Heart Association (AHA) and the National Heart, Lung, and Blood Institute (NHLBI). Body mass index (BMI) was calculated for all patients and categorized according to standard definitions. Glycemic status was assessed using hemoglobin A1c (HbA1c) levels in place of fasting glucose to improve data completeness and reliability in real-world clinical records. Additional cardiometabolic components included hypertension (HTN), chronic kidney disease (CKD), reduced high-density lipoprotein cholesterol (HDL < 50 mg/dL), and elevated triglycerides (> 150 mg/dL). Patients meeting criteria for three or more of these cardiometabolic abnormalities were classified as having metabolic syndrome. Comorbidity ascertainment incorporated a multimodal approach including ICD-10 diagnostic codes, clinician documentation within the electronic medical record, and laboratory values meeting predefined diagnostic thresholds.

Detailed treatment information for the Keynote-522 regimen was collected, including chemotherapy and immunotherapy dosing, schedules, and completion status. Adverse events (AEs) during treatment were recorded and graded according to the CTCAE, version 8.0.

The primary outcomes were pCR and OS. pCR was chosen as a validated surrogate marker for long-term outcomes in TNBC, and OS was selected as the ultimate patient-relevant endpoint. The impact of baseline cardiometabolic comorbidities on treatment tolerability was also assessed.

Missing data were handled by complete-case analysis; patients with missing values for covariates of interest were excluded from multivariable models, and no imputation was performed.

Follow-up time was calculated from the date of diagnosis until death or the last documented clinical encounter. Patients alive at the last follow-up were censored at that time. The median follow-up duration was 28.8 months.

### Statistical analysis

Patient characteristics were summarized by overall metabolic syndrome status and pathological complete response status. Fisher’s exact test was used to compare metabolic syndrome status and pCR status between patient groups. Overall survival (OS) after diagnosis and after treatment completion were was estimated using the Kaplan-Meier method and compared using the log-rank test. The Cox proportional hazards model was used to assess the association between patient and clinical factors and OS. The backward elimination procedure was used to identify final models with only significant factors, starting from all factors of interest. All tests were two-sided and p-values of 0.05 or less were considered statistically significant. Statistical analysis was carried out using SAS Studio 3.7 (SAS Institute, Cary, NC) and R version 4.4 (R Foundation, Vienna, Austria).

## Results

Of the 222 patients with early-stage TNBC treated with the Keynote-522 regimen, (24.8%) met criteria for metabolic syndrome (Table 1). The median age at diagnosis was higher among patients with metabolic syndrome at 65 vs. 55 years old (*p* = 0.0004) (Supplemental Table 1).

Racial and ethnic differences were observed. Black patients comprised 43.64% of those with metabolic syndrome compared to 17.96% without. Conversely, White patients were less likely to have metabolic syndrome (47.24% vs. 74.45%) (Table 1).

**Table 1:** Stratification of baseline patient characteristics by metabolic syndrome status

	Metabolic Syndrome			
	No		Yes	
	*N*	%	*N*	%
Total	167	100	55	100
Age at Diagnosis				
< 50	59	35.33	12	21.82
>=50	108	64.67	43	78.18
Race				
Unknown	2	1.2	2	3.64
American Indian/Alaska native	1	0.6	0	0
Asian	3	1.8	0	0
Black	30	17.96	24	43.64
Hispanic	4	2.4	2	3.64
White	126	75.45	26	47.27
Multiracial	1	0.6	1	1.82
Stage				
I	12	7.19	4	7.27
II	79	47.31	30	54.55
III	76	45.51	21	38.18
Smoking				
No	110	65.87	34	61.82
Yes	57	34.13	21	38.18
BRCA				
No	152	91.02	47	85.45
Yes	15	8.98	8	14.55
Vital				
Alive	156	93.41	41	74.55
Dead	11	6.59	14	25.45
Discontinuation of Treatment				
No	113	67.66	39	70.91
Yes	54	32.34	16	29.09
Dose Reduction				
No	133	79.64	37	67.27
Yes	34	20.36	18	32.73
Pathological Complete Remission				
No	52	31.14	15	27.27
Yes	115	68.86	40	72.73
Disease-specific survival status				
No	167	100	53	96.36
Yes	0	0	2	3.64
Disease Relapse				
No	166	99.4	53	96.36
Yes	1	0.6	2	3.64

**Table 2:** Stratification of baseline patient cardiometabolic co-morbidities

	Metabolic Syndrome			
	No		Yes	
	*N*	%	*N*	%
Total	167	100	55	100
CKD				
No	158	94.61	43	78.18
Yes	8	4.79	12	21.82
HTN				
No	126	75.45	17	30.91
Yes	41	24.55	38	69.09
Obesity				
No	107	64.07	8	14.55
Yes	60	35.93	47	85.45
DM				
No	154	92.22	15	27.27
Yes	13	7.78	40	72.73
PCOS				
No	166	99.4	53	96.36
Yes	1	0.6	2	3.64
HDL < 50				
Unknown	24	14.37	1	1.82
No	123	73.65	8	14.55
Yes	20	11.98	46	83.64
TAGs > 150				
Unknown	24	14.37	2	3.64
No	133	79.64	21	38.18
Yes	10	5.99	32	58.18
NASH				
No	164	98.2	52	94.55
Yes	3	1.8	3	5.45

**Table 3:** Stratification of baseline patient cardiometabolic co-morbidities by immune-related adverse effects

	Metabolic Syndrome				*p*-value
	No		Yes		
	*N*	%	*N*	%	
Immune Related Adverse Effect Grade					
0	126	75.45	45	81.82	0.88
1	7	4.19	2	3.64	
2	28	16.77	6	10.91	
3	6	3.59	2	3.64	
irAE related hospitalization					
N/A	126	75.45	45	81.82	
No	27	16.17	8	14.55	0.47
Yes	14	8.38	2	3.64	
irAE related treatment intervention					
N/A	126	75.45	45	81.82	0.67
No	8	4.79	3	5.45	
Yes	33	19.76	7	12.73	

Obesity was more prevalent in the metabolic syndrome group (≥ 30 kg/m²: 85.45% vs. 35.93%). Diabetes was also more common among patients with metabolic syndrome (72.73% vs. 7.78% for diabetes) (Table 2).

Hypertension was observed in 69.09% of patients with metabolic syndrome compared to 24.55% without. Chronic kidney disease (CKD) was more frequent in the metabolic syndrome group (21.82% vs. 4.79%). Lipid abnormalities were significantly more common: 83.64% had low HDL (< 50 mg/dL) and 58.18% had elevated triglycerides (> 150 mg/dL), compared to 11.98% and 5.99%, respectively, in patients without metabolic syndrome (both *p* < 0.001). Rates of non-alcoholic steatohepatitis (NASH) and polycystic ovary syndrome (PCOS) were low across both groups (Table 2).

Regarding treatment tolerability, patients with metabolic syndrome had higher rates of chemotherapy dose reductions due to adverse events (32.73% vs. 20.36%). However, rates of treatment discontinuation due to toxicity were similar (29.09% vs. 32.34%) (Table 1). No significant associations were observed between immune-related adverse events, hospitalizations, or treatment interventions in patients with metabolic syndrome (Table 3).

### Pathological response

Among the 222 patients with evaluable pathologic response, 155 patients (69.82%) achieved a pathologic complete response (pCR), while 67 patients (20.18%) did not (Table 4).

**Table 4:** Summary of patient characteristics stratified by pathologic complete response

	Pathological Complete Response				*P*-value
	*N*		Y		
	*N*	%	*N*	%	
Age at DX					
< 50	17	25.37	54	34.84	0.21
>=50	50	74.63	101	65.16	
Metabolic Syndrome					
No	52	77.61	115	74.19	0.62
Yes	15	22.39	40	25.81	
CKD					
Unknown	0	0	1	0.65	
No	59	88.06	142	91.61	0.32
Yes	8	11.94	12	7.74	
HTN					
No	40	59.7	103	66.45	0.36
Yes	27	40.3	52	33.55	
Obesity					
No	37	55.22	78	50.32	0.56
Yes	30	44.78	77	49.68	
DM					
DM	11	16.42	24	15.48	0.77
Normal	42	62.69	104	67.1	
Pre-DM	14	20.9	27	17.42	
PCOS					
No	67	100	152	98.06	0.56
Yes	0	0	3	1.94	
HDL < 50					
Unknown	8	11.94	17	10.97	
No	40	59.7	91	58.71	0.87
Yes	19	28.36	47	30.32	
TAGs > 150					
Unknown	8	11.94	18	11.61	
No	49	73.13	105	67.74	0.35
Yes	10	14.93	32	20.65	
NASH					
No	67	100	149	96.13	0.18
Yes	0	0	6	3.87	

No statistically significant association was found between metabolic co-morbidities and pathological complete response (Table 5).

In the multivariable logistic regression model evaluating predictors of pCR, a backward-elimination procedure was used to identify significant covariates. None were identified.

### Overall Survival

The median follow-up among those alive was 28.8 months (range: 3.4–54.2 months). Median overall survival (OS) was 113.9 months (95% CI: 52.8-NA). The estimated 1-year and 2-year OS rates for the overall cohort were 95% (95% CI: 93–98%) and 93% (95% CI: 90–96%), respectively. Breast cancer-specific survival could not be analyzed statistically because only 2 events occurred during this time, and the other 23 deaths were secondary to cardiac or infection-related events.

**Fig. 1:**
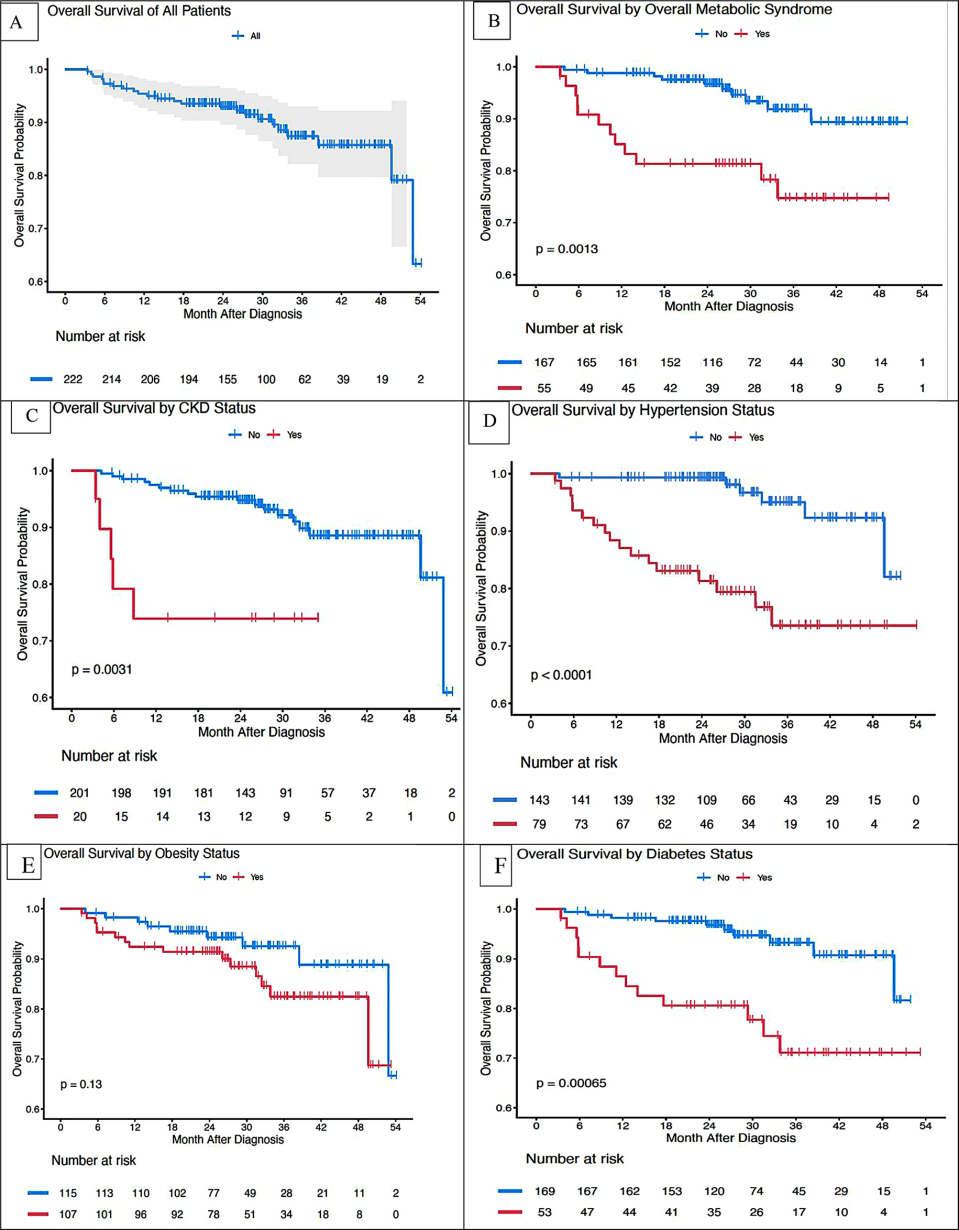
Kaplan–Meier overall survival curves stratified by clinical and metabolic factors. (**A**) All Patients (**B**) presence of metabolic syndrome, (**C**) CKD Status, (**D**) Hypertension Status (**E**) Obesity Status (**F**) Diabetes status

Patients with metabolic syndrome had significantly worse OS than those without (2-year OS rates: 0.81 (95% CI: 0.72–0.92) vs. 0.97 (0.94-1), *p* = 0.0013) (Supplemental Table 2, Fig. 1). Similarly, patients with chronic kidney disease (2-year OS rates: 0.74 (0.57–0.96) vs. 0.95 (0.92–0.98), *p* = 0.0031), hypertension (2-year OS rates: 0.81(0.73–0.91) vs. 0.99 (0.98-1), *p* < 0.0001), and diabetes mellitus (2-year OS rates: 0.81(0.70–0.92) vs. 0.97 (0.94-1), *p* = 0.0006) experienced significantly lower survival (Supplemental Table 2, Fig. 1). A multivariable Cox proportional hazards model was constructed using a backward elimination procedure, starting with all variables significant in univariate analyses.

After adjustment for age, hypertension remained significantly associated with worse overall survival. Patients with hypertension had over four times the risk of death compared to those without hypertension (HR = 3.83; 95% CI: 1.47–9.96; *p* = 0.006) and diabetes mellitus (HR 3.07, 95% CI: 1.33–7.08, *p* = 0.009 (Table 5).

**Table 5:** Summary of multivariate Cox proportional hazards model results for overall survival (OS) after treatment to assess treatment discontinuation and dose reduction effects on Overall Survival

Factor	Comparison	Hazard Ratio	95% LCL	95% UCL	*P*-value
Age at DX	>=50 vs. <50	0.893	0.315	2.531	0.83
HTN	Yes vs. No	3.832	1.474	9.962	0.006
DM	Yes vs. No	3.071	1.332	7.082	0.009

## Discussion

In this retrospective cohort study of early-stage TNBC patients treated with the Keynote-522 regimen, approximately one-quarter (24.7%) met the criteria for metabolic syndrome. Metabolic co-morbidities were not significantly associated with pathological response. In contrast, cardiometabolic comorbidities were strongly associated with overall survival. Among individual components, hypertension was the strongest independent predictor of mortality, adjusted for age. Hypertensive patients had a significantly higher risk of death (HR 3.83, 95% CI 1.47–9.96, *p* = 0.006) and diabetes mellitus (HR 3.07, 95% CI 1.33–7.98, *p* = 0.009). Chronic kidney disease and obesity were also associated with worse OS.

These findings support the need for systematic cardiometabolic risk screening at the time of TNBC diagnosis. Hypertension and diabetes mellitus control may represent a modifiable prognostic factor even in the context of chemoimmunotherapy.

### Metabolic syndrome and pathological response

The high prevalence of metabolic syndrome in our cohort underlines the cardiometabolic burden among patients with early-stage TNBC. The absence of an association between comorbidities and pCR, despite their strong effect on OS, may reflect mechanisms beyond tumor response, including treatment tolerance, systemic inflammation, and competing risks of non-cancer mortality.

### Survival Impact of cardiometabolic comorbidities

The impact of comorbidities on OS in patients with MBC has been well documented [17, 18]. A systematic review and meta-analysis of 42,135 breast-cancer survivors found that metabolic syndrome increased the risk of recurrence (HR 1.69), breast-cancer mortality (HR 1.83), and shorter disease-free survival (HR 1.57) [20]. Prior studies in MBC have shown that hypertension is independently associated with reduced survival, with hazard ratios of 1.45 (95% CI, 1.12–1.89) and 1.33 (95% CI, 1.07–1.67) in separate cohorts [18, 19]. However, this association has not been previously characterized in early-stage TNBC treated with neoadjuvant chemotherapy and immunotherapy.

In our study, several clinical and cardiometabolic factors were significantly associated with OS. Patients with metabolic syndrome had worse 2-year OS compared to those without (HR 0.81, 95% Confidence Interval (CI) 0.72–0.92; *p* = 0.0013 (Supplemental Table 1, Table 5; Fig. 1B), highlighting the prognostic relevance of cardiometabolic health. Among individual components, hypertension and diabetes mellitus had the strongest association: hypertensive patients had lower 2-year OS (HR 0.81, 95% CI 0.73–0.91, *p* < 0.0001), and in multivariable analysis, it remained independently associated with reduced survival (HR 3.83, 95% CI 1.47–9.96, *p* = 0.006). Patients with diabetes mellitus also had a lower 2-year OS (HR 0.81, 95% CI 0.73–0.92, *p* < 0.0006) and an independent association with (HR 3.07, 95% CI 1.33–7.98, *p* = 0.009). This effect size is comparable to the prognostic impact of residual disease following neoadjuvant therapy, underscoring that comorbidities carry prognostic weight like established tumor-related risk factors.

Health equity considerations are also critical. In our cohort, Black women were disproportionately affected by metabolic syndrome compared with White women. This finding reflects broader disparities in TNBC outcomes and highlights the intersection between comorbidities, race, and survival inequities. Addressing cardiometabolic health may therefore represent a key opportunity to reduce disparities in treatment and care for TNBC.

Multiple pathophysiological mechanisms may explain the link between hypertension and adverse outcomes. Chronic inflammation contributes to both hypertension and cancer progression through cytokine dysregulation, endothelial dysfunction, impaired apoptosis, and increased cellular proliferation [21–24]. Elevated vascular endothelial growth factor (VEGF), implicated in both hypertension and breast cancer, promotes tumor angiogenesis, vascular permeability, and metastasis and is associated with reduced progression-free and overall survival [25, 26]. Shared biological pathways, including hormonal dysregulation, physical inactivity, and endothelial dysfunction, may further contribute to poor outcomes across hypertension, cardiovascular disease, and breast cancer [27, 28].

Diabetes and metabolic syndrome were also significantly associated with reduced OS (Supplemental Table 1). These findings align with previous studies showing that both conditions are linked to increased mortality in breast cancer. A meta-analysis of 17 studies involving 48,315 women found that diabetes was associated with a 49% increased risk of all-cause mortality (HR = 1.49; 95% CI: 1.35–1.65) [29]. Similarly, a systematic review and meta-analysis of 42,135 patients reported that metabolic syndrome increased breast cancer mortality by 83% (95% CI: 1.35–2.49) [20]. Both conditions are characterized by hyperinsulinemia, chronic inflammation, and hormonal imbalances that may contribute to tumor progression and reduced survival [30].

CKD was also associated with significantly worse OS (2-year OS: HR 0.74, 95% CI 0.57–0.96, *p* = 0.0031). Despite its clinical relevance, patients with CKD are often excluded from oncology trials, even though the kidneys excrete nearly half of all chemotherapeutic agents [31–33]. The altered pharmacokinetics in this population may increase the risk of toxicity. For instance, trastuzumab has been associated with increased risk of cardiotoxicity in patients with CKD, likely due to direct effects on cardiomyocytes [34, 35].

Taken together, these findings underscore the importance of incorporating comorbid conditions into both the clinical management and prognostic assessment of patients with breast cancer. High-risk patients, including those with hypertension, diabetes, CKD, and metabolic syndrome, experience poorer outcomes and may benefit from coordinated care involving primary care providers, cardiologists, endocrinologists, and nephrologists.

### Limitations

Our study has several limitations. First, its retrospective design and single-institution setting may limit generalizability. Second, the relatively short median follow-up of 28.8 months may underestimate long-term mortality and recurrence risks. Third, residual confounding by unmeasured variables such as treatment adherence, socioeconomic status, and tumor biology cannot be excluded. We also did not assess longitudinal changes in blood pressure, glycemic control, or lipid levels during therapy, which may further influence outcomes.

### Future directions

Prospective, multi-institutional studies with longer follow-up are needed to validate these findings. Future research should integrate detailed cardiometabolic phenotyping, including blood pressure trajectories, glycemic control, lipid profiles, and inflammatory biomarkers, with immune profiling to better understand how metabolic dysfunction influences tumor biology and response to immunotherapy. Randomized interventional studies are also warranted. Potential strategies include aggressive hypertension management, particularly with ACE inhibitors or ARBs, which may have anti-tumor properties, alongside novel metabolic-modifying agents such as GLP-1 receptor agonists or SGLT2 inhibitors. Structured lifestyle interventions targeting obesity, physical inactivity, and diet during chemo-immunotherapy may further enhance treatment tolerance and survival outcomes.

## Conclusions

Our findings demonstrate that cardiometabolic health, particularly hypertension and diabetes mellitus, is as prognostically relevant as traditional tumor factors in early-stage TNBC. While metabolic syndrome did not influence pCR, it was associated with worse overall survival, with hypertension and diabetes mellitus, emerging as an independent predictor of mortality. Incorporating systematic cardiometabolic risk assessment and proactive management into oncology care pathways may improve treatment tolerance and survival outcomes. Addressing modifiable comorbidities represents an actionable strategy to optimize outcomes and reduce disparities in TNBC care.

## Supplementary Information

Below is the link to the electronic supplementary material.

Supplementary Material 1

## Data Availability

No datasets were generated or analysed during the current study.

## Abbreviations

AHA: American Heart Association
AE: Adverse Event
ARB: Angiotensin II Receptor Blocker
ARNI: Angiotensin Receptor–Neprilysin Inhibitor
BC: Breast Cancer
BMI: Body Mass Index
BRCA: Breast Cancer Gene
CI: Confidence Interval
CKD: Chronic Kidney Disease
CTCAE: Common Terminology Criteria for Adverse Events
DM: Diabetes Mellitus
ER: Estrogen Receptor
GLP-1: Glucagon-Like Peptide-1
HDL: High-Density Lipoprotein
HER2: Human Epidermal Growth Factor Receptor 2
HR: Hazard Ratio
HTN: Hypertension
HbA1c: Hemoglobin A1c
ICD-10: International Classification of Diseases, 10th Revision
ICI: Immune Checkpoint Inhibitor
irAE: Immune-related Adverse Event
NASH: Non-Alcoholic Steatohepatitis
NHLBI: National Heart, Lung, and Blood Institute
OS: Overall Survival
PCOS: Polycystic Ovary Syndrome
PD-L1: Programmed Death Ligand 1
pCR: Pathologic Complete Response
PR: Progesterone Receptor
SGLT2: Sodium-Glucose Cotransporter-2
TAG: Triglycerides
TIL: Tumor-Infiltrating Lymphocytes
TNBC: Triple-Negative Breast Cancer
