# Supplementary material for: Impact of cardiometabolic health on treatment outcomes in early-stage triple-negative breast cancer receiving chemoimmunotherapy
Source: Breast Cancer Res Treat. 2026 Jun 12;217(2):50. doi: 10.1007/s10549-026-07995-5 (PMC13263220; doi:10.1007/s10549-026-07995-5)
Supplement: Supplementary file 1 — Supplementary Material 1 [file 10549_2026_7995_MOESM1_ESM.docx]

Supplemental Table 1: **Distribution of Age at Diagnosis and Number of Treatment Cycles Stratified by Metabolic Syndrome**

|  | | **N** | **Min** | **25th %ile** | **Median** | **75th %ile** | **Max** | **P-value** |
| --- | --- | --- | --- | --- | --- | --- | --- | --- |
| **Age at Diagnosis** | **Metabolic Syndrome** |  |  |  |  |  |  |  |
|  | **No** | 167 | 23 | 44 | 55 | 64 | 90 | 0.0004 |
|  | **Yes** | 55 | 30 | 51 | 65 | 70 | 85 |  |
|  | **All** | 222 | 23 | 47 | 58 | 68 | 90 |  |
| **Number of Total Cycles** | **Metabolic Syndrome** |  |  |  |  |  |  |  |
|  | **No** | 167 | 1 | 5 | 9 | 12 | 18 | 0.74 |
|  | **Yes** | 55 | 1 | 6 | 9 | 12 | 16 |  |
|  | **All** | 222 | 1 | 5 | 9 | 12 | 18 |  |

Supplemental Table 2: Summary of overall survival (OS) after diagnosis by patient characteristics.


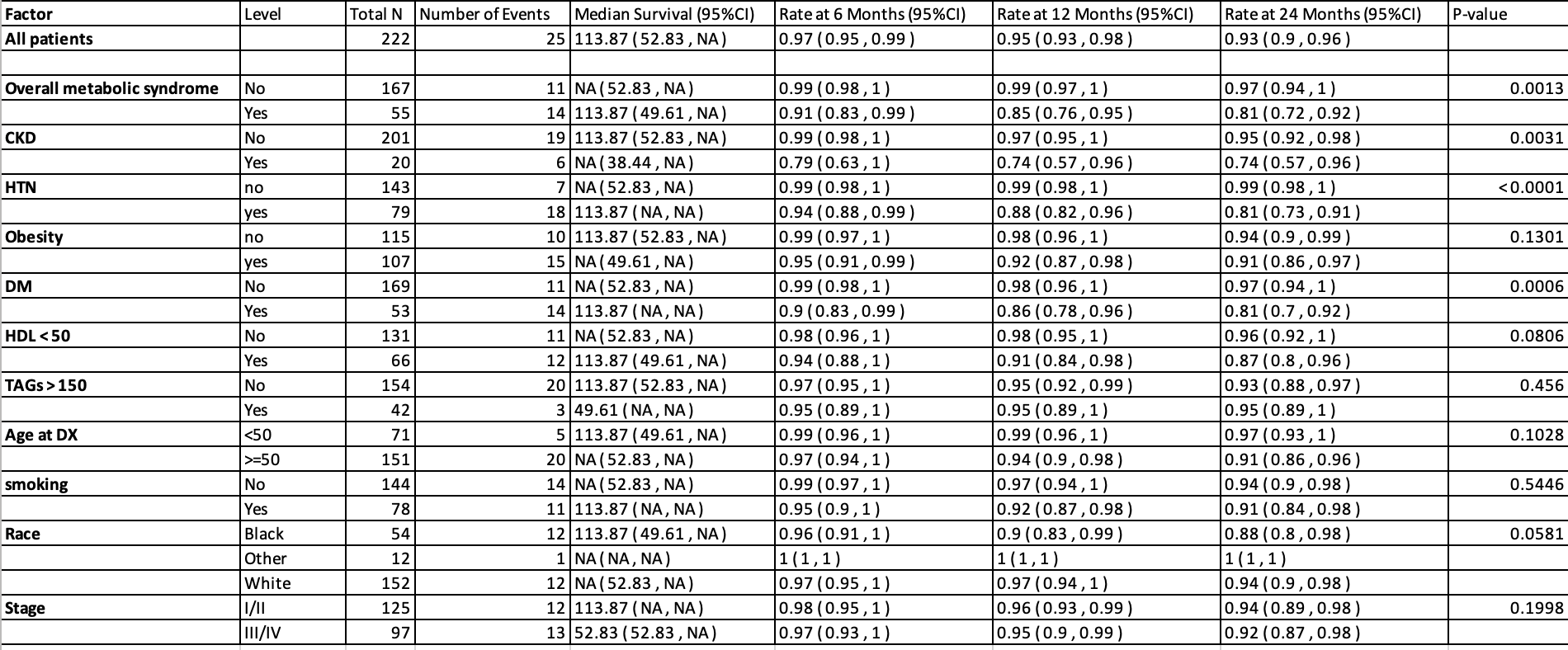


Supplementary Table 3: Medication Classes by Therapeutic Category Among Patients with Metabolic Co-morbidities

| **Therapeutic Category** | **Medication Class** | **Medications** |
| --- | --- | --- |
| Antihypertensive Agents | ACE inhibitors | lisinopril, ramipril |
| Antihypertensive Agents | ARBs | losartan, valsartan, olmesartan, irbesartan, telmisartan |
| Antihypertensive Agents | ARB combinations | losartan-hydrochlorothiazide, valsartan-hydrochlorothiazide, telmisartan-amlodipine |
| Antihypertensive Agents | Beta blockers | metoprolol, metoprolol succinate ER, carvedilol, atenolol, propranolol |
| Antihypertensive Agents | Calcium channel blockers | amlodipine, nifedipine |
| Antihypertensive Agents | Diuretics | hydrochlorothiazide, chlorthalidone, spironolactone |
| Antihypertensive Agents | Vasodilators | hydralazine |
| Antihypertensive Agents | ARNI | sacubitril-valsartan |
| Lipid-Lowering Therapy | Statins | atorvastatin, rosuvastatin, simvastatin, pravastatin, lovastatin |
| Lipid-Lowering Therapy | Cholesterol absorption inhibitor | ezetimibe |
| Glucose-Lowering Therapy | Biguanide | metformin |
| Glucose-Lowering Therapy | Sulfonylureas | glipizide, glimepiride |
| Glucose-Lowering Therapy | SGLT2 inhibitors | dapagliflozin, empagliflozin |
| Glucose-Lowering Therapy | GLP-1 receptor agonists | semaglutide, dulaglutide |
| Glucose-Lowering Therapy | Dual GIP/GLP-1 agonist | tirzepatide |
| Glucose-Lowering Therapy | Insulin therapy | insulin glargine, insulin lispro, Humulin, insulin pump |
| Combination Antihypertensive Therapy | Fixed-dose combinations | amlodipine-benazepril, lisinopril-hydrochlorothiazide |
